# The Association of Serum Total Bile Acids With Bone Mineral Density in Chinese Adults Aged 20–59: A Retrospective Cross-Sectional Study

**DOI:** 10.3389/fendo.2022.817437

**Published:** 2022-04-19

**Authors:** Jingxin Liu, Yuxing Chen, Qi Luo

**Affiliations:** ^1^ Department of Orthopedics Ward 1, Hainan Western Central Hospital, Danzhou, China; ^2^ Department of Spine Surgery, Ganzhou People’s Hospital, Ganzhou, China

**Keywords:** serum total bile acids, bone mineral density, osteoporosis, Chinese adults, retrospective cohort study

## Abstract

**Objective:**

According to a recent study, serum total bile acids (TBA) may preserve lumbar bone mineral density (BMD) in Cushing syndrome patients, and BMD is directly linked to bone health. We were interested in examining the association between TBA and in Chinese adults aged 20–59 years.

**Methods:**

We retrospectively analyzed the physical examination results of 2,490 general healthy subjects in Hainan West Central Hospital. Femoral neck BMD and TBA were measured, and the relationship between TBA and femoral neck BMD was evaluated by curve fitting, a generalized additive model, and multiple linear regression analysis.

**Results:**

The fitted smooth curve and generalized additive model showed a nonlinear relationship between TBA and femoral neck BMD, and a positive correlation between TBA and femoral neck BMD was found after we made adjustments for the potential confounders.

**Conclusion:**

TBA is positively associated with femoral neck BMD in Chinese adults aged 20–59 years.

## Introduction

Osteoporosis is the most common bone disease, which is characterized by a decrease in bone mineral density (BMD) and quality for various reasons (1, 2). It is estimated that the prevalence of osteoporosis in China in 2015 was 5.30% (95% CI, 2.23%–11.85%) for men and 25.94% (95% CI, 15.42%–39.69%) for women, which has brought a huge economic burden to the society (3). BMD is the major index of bone health and has advantages in predicting osteoporotic fractures (4, 5).

Bile acids are major components of bile and play an essential role in glycolipid metabolism and regulation of the intestinal flora (6–8). Recent studies have found that bile acids are closely related to bone metabolism. Vitamin D in blood circulation is positively correlated with bile acid (9), which can promote intestinal absorption of fat-soluble vitamin D, improve BMD, and reduce the fracture rate (10–12). In addition, bile acids affect bone metabolism and BMD by activating the farnesoid X receptor (FXR) and G protein-coupled bile acid receptor 5 (TGR5) signaling pathways (13, 14). However, to date, only a few clinical studies have demonstrated an association between TBA and BMD. To evaluate the association between TBA and BMD in the Chinese population aged 20–59 years, we retrospectively analyzed general physical examination population data provided by the physical examination institution of the Western Central Hospital in Hainan Province, China, and studied the relationship between serum bile acid and BMD at the proximal femur.

## Methods

### Study Sample and Variable Collection

**Figure 1:**
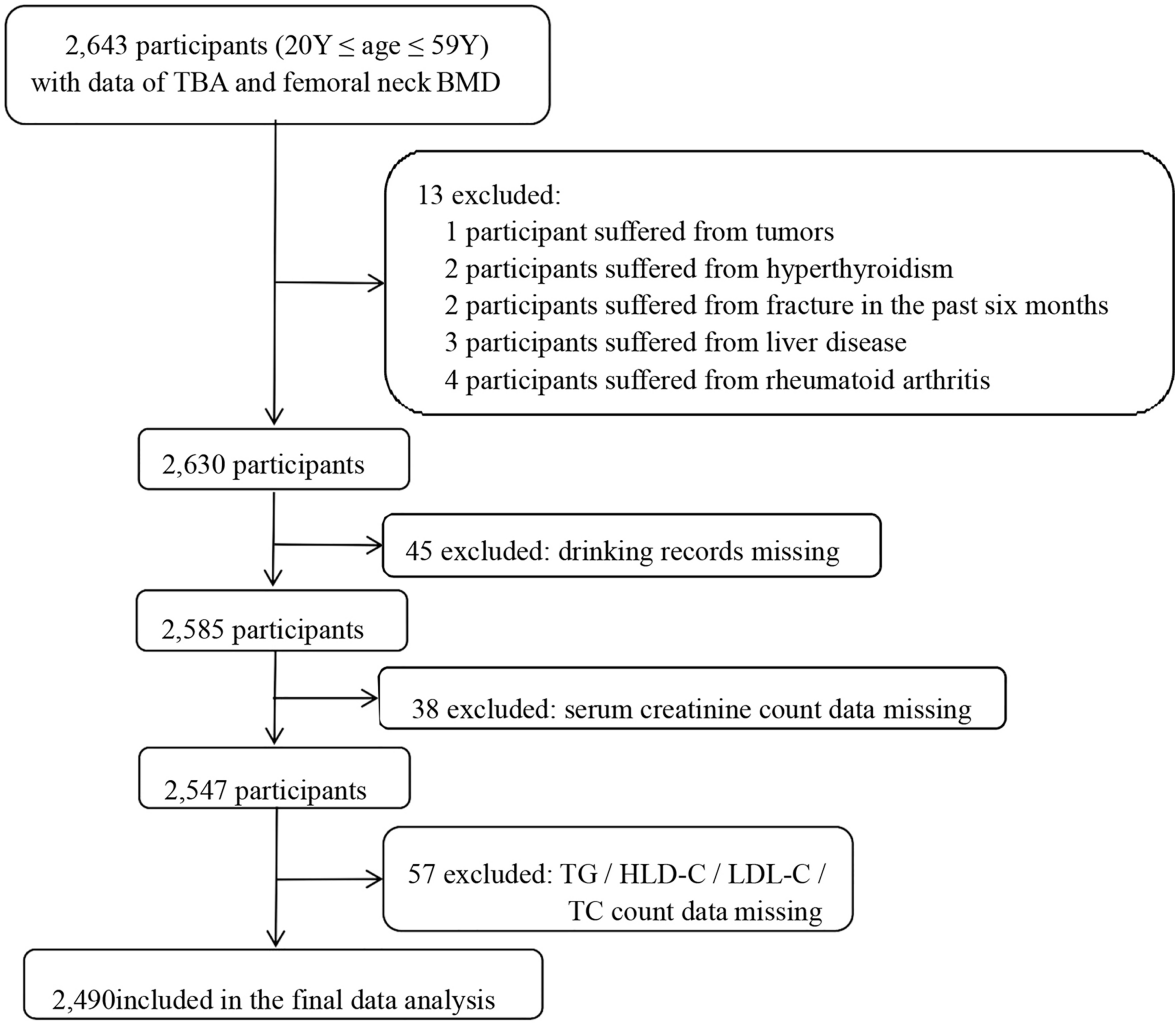
Flow chart showing the selection process of the study population.

This was a retrospective cross-sectional study. The in-service staff who took the physical examination in the health examination center of Hainan West Central Hospital from January 2018 to December 2020 were recruited to our study. Inclusion criteria were as follows: (1) 20≤ age ≤59 years old and (2) completion of femoral neck BMD and TBA examinations. Exclusion criteria: (1) self-reported history of disease (liver disease, tumors, rheumatoid disease, hyperthyroidism, and chronic infection) and (2) participants with incomplete study data. In total, 2,490 participants were then included in this study. The selection process of the study sample is shown in **Figure 1**.

This study has been authorized by the research ethics committee of Hainan West Central Hospital and was performed according to the principles outlined in the Declaration of Helsinki. Because this investigation was a retrospective analysis, the hospital’s institutional review board waived the participants written informed consent requirement.

Femoral neck BMD was measured by DEXA scanning though a GE Lunar Prodigy Advance PA + 300164 (Lunar Corp, Adison, WI, USA). BMD was determined according to the criteria of lunar protocols and measured at the midpoint of the femur and 2 cm proximally of the femoral head, and the calculation method of femoral neck BMD (g/cm^2^) was bone mineral content (BMC) (g)/bone area (cm^2^).

Serum was collected at 8 h after fasting and TBA, aspartate aminotransferase (AST), alanine transaminase (ALT), alkaline phosphatase (ALP), glutamyl transpeptidase (GGT), triglyceride (TG), high-density lipoprotein cholesterol (HLD-C), low-density lipoprotein cholesterol (LDL-C), total cholesterol (TC), total bilirubin (TBil), and serum creatinine were determined on the Hitachi Chemical Analyzer 7600 using a reagent (PureautoSTBA, SEKISUI Medical Co., Ltd, Hitachi Coro, Tokyo, Japan) using enzyme cycling. Age, sex, education level (less than high school, high school or above), smoking status (smoking status was defined as currently smoking 10 cigarettes a day for more than half a year), drinking status (drinking status was defined as continuous drinking for more than 5 years, with men drinking 40 g/day and women drinking 20 g/day), and medical history of hypertension and diabetes were collected through questionnaires. Height and weight were also collected. Age group defined as <35 and ≥35 years old. Body mass index (BMI) was calculated as weight (kg)/height (m) squared. Medical history of diabetes and hypertension was obtained from inpatient medical records. Patients were defined as obese with a BMI ≥30 (14). The glomerular filtration rate in serum creatinine, eGFR (ml/min/1.73 m^2^) = 175 × standardized Scr^−1.234^ × age^−0.179^ × 0.79 (in female), and eGFR <60 ml/min/1.73 m^2^ were classified as chronic kidney disease (CKD) (15, 16).

### Statistical Analysis

Study participants were divided into four groups based on quartiles of TBA. If the data were normally distributed, the continuous variable was expressed as the mean standard deviation. If the data were skewed, the continuous variable was expressed as the median of the interquartile range (Q1–Q3). The variable was expressed as a number (percentage). The Wilcoxon or Kruskal–Wallis tests were taken to analyze group differences for continuous variables and the Chi-square test for categorical variables. In addition, smooth curve fitting and generalized additive model were used to solve the nonlinear relationship between TBA and femoral neck BMD. Three models were fitted: model 1 was unadjusted; model 2 was adjusted for age and sex; and model 3 was adjusted for variables including sex, age, education, smoking status, obesity, TBil, AST, ALP, GGT, ALT, TC, TG, HDL-C, and LDL-C. *p*-values for trend were calculated with the quartile of TBA as an ordinal variable. Subgroup analyses were performed to assess the robustness of the association between TBA and femoral neck BMD based on sex, education level, smoking status, drinking status, obesity, hypertension, diabetes, and CKD. We used the statistical software packages R (http://www.R-project.org) and Empowerstats (http://www.Empowerstats.com, X&Y Solutions, Inc., Boston, MA). We defined *p*-values of less than 0.05 (bilateral) as statistically significant.

## Results

### Clinical Characteristics

The clinical characteristics of participants in the TBA quartile subgroup and overall population are presented in **Table 1**. It contained 2,490 participants, whose average age was 38.83 ± 12.1 years old, and there were 1,331 female participants. The median femoral neck BMD was 0.8 (interquartile range, 0.8–0.9) g/cm^2^, and the median femoral neck BMD of TBA was 8.5 (interquartile range, 8.0–9.0) µmol/L. The femoral neck BMD levels increased significantly with the increase in TBA quartile, from 0.8 (interquartile range, 0.7–0.9) g/cm^2^ in quartiles Q1 to 0.9 (interquartile range, 0.8–1.0) g/cm^2^ in Q4. Age, smoking status, drinking status, diabetes, TG, and TBil increased as the TBA quartiles increased.

**Table 1:** Clinical characteristics of the participants according to quartiles of TBA.

Characteristics	Overall mean ± SD/N (%)	Quartiles of total bile acids				*p*-value
		Q1	Q2	Q3	Q4	
*N*	2,490	624	623	621	622	–
Age (years)	38.8 ± 12.1	38.5 ± 12.6	39.1 ± 11.7	38.4 ± 12.1	39.3 ± 12.0	0.447
Age ≥35 years [n (%)]	1,488 (59.8%)	359 (57.5%)	380 (61.0%)	364 (58.6%)	385 (61.9%)	0.361
Women [n (%)]	1,331 (53.5%)	336 (53.9%)	360 (58.0%)	394 (63.3%)	336 (53.9%)	<0.001
More high school [n (%)]	1,598 (64.2%)	383 (61.4%)	410 (65.8%)	410 (66.0%)	395 (63.5%)	0.273
Obesity [n (%)]	260 (10.4%)	120 (19.2%)	61 (9.8%)	42 (6.8%)	37 (6.0%)	<0.001
Smoking status [n (%)]	792 (31.8%)	240 (38.5%)	191 (30.7%)	201 (32.4%)	160 (25.7%)	<0.001
Drinking status [n (%)]	61 (2.5%)	27 (4.3%)	14 (2.3%)	11 (1.8%)	9 (1.5%)	0.004
Diabetes [n (%)]	94 (3.8%)	48 (7.7%)	20 (3.2%)	16 (2.6%)	10 (1.6%)	<0.001
Hypertension [n (%)]	604 (24.3%)	204 (32.7%)	161 (25.8%)	117 (18.8%)	122 (19.6%)	<0.001
CKD [n (%)]	96 (3.9%)	27 (4.3%)	24 (3.9%)	20 (3.2%)	25 (4.0%)	0.778
TBil (IU/ml)	0.5 (0.4–0.7)	0.5 (0.4–0.7)	0.5 (0.4–0.7)	0.5 (0.4–0.7)	0.50 (0.40–0.70)	0.224
AST (IU/ml)	23.0 (19.0–31.0)	23.0 (18.0–28.0)	23.0 (18.0–28.0)	23.0 (19.0–28.0)	23.0 (19.0–31.0)	0.002
ALP (IU/ml)	54.0 (45.0–65.0)	51.00 (41.0–61.0)	48.00 (40.0–58.0)	49.00 (40.0–59.0)	54.0 (45.0–65.0)	<0.001
GGT (IU/ml)	10.0 (6.0–16.0)	8.00 (5.0–12.0)	7.0 (5.0–10.0)	6.0 (5.0–10.0)	10.0 (6.0–16.0)	<0.001
ALT (IU/ml)	36.3 (30.0–42.6)	33.9 (27.6–39.0)	30.9 (25.8–36.6)	29.1 (24.3–34.5)	36.3 (30.0–42.6)	<0.001
TC (mg/dl)	174.0 (153.0–197.0)	177.0 (159.0–198.5)	173.0 (153.0–195.0)	175.0 (155.0–201.0)	174.0 (153.0–197.0)	0.15
TG (mg/dl)	69.0 (63.0–75.3)	69.0 (63.0–75.0)	68.0 (62.0–75.0)	69.0 (63.0–75.0)	69.0 (63.0–75.3)	0.283
HDL-C (mg/dl)	52.0 (44.0–61.0)	52.0 (44.0–60.0)	52.0 (44.0–61.0)	52.0 (44.0–62.0)	52.0 (44.0–61.0)	0.807
LDL-C (mg/dl)	107.0 (86.0–128.0)	110.0 (93.0–128.0)	105.0 (85.0–128.0)	108.0 (89.0–128.0)	107.0 (86.0–128.0)	0.139
TBA (µmol/L)	8.5 (8.0–9.0)	7.5 (7.2–7.8)	8.2 (8.1–8.4)	8.7 (8.6–8.8)	9.25 (9.1–9.5)	<0.001
Femoral neck BMD (g/cm^2^)	0.8 (0.8–0.9)	0.8 (0.7–0.9)	0.8 (0.8–0.9)	0.9 (0.8–0.9)	0.9 (0.8–1.0)	<0.001

CKD, chronic kidney diseases; ALT, alanine transaminase; AST, aspartate aminotransferase; ALP, alkaline phosphatase; GGT, glutamyl transpeptidase; HDL-C, high-density lipoprotein cholesterol; LDL-C, low-density lipoprotein cholesterol; TBA, total bile acids; TBIL, total bilirubin; TC, total cholesterol; TG, triglyceride; BMD, bone mineral density.

### The Relevance Between Serum Bile Acid and Lumbar Bone Mineral Density

**Figure 2:**
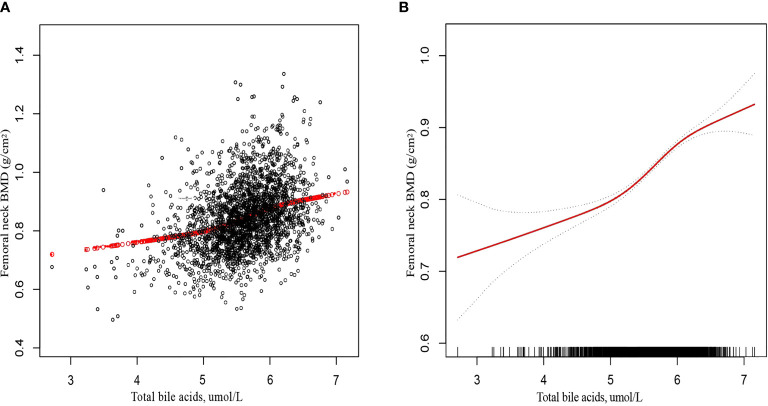
The association between total bile acids and femoral neck bone mineral density. **(A)** Each black point represents a sample. **(B)** The solid red line represents the smooth curve fit between variables. Gray bands represent the 95% confidence interval from the fit. Age, sex, education, obesity, smoking status, CKD, diabetes, hypertension, drinking status, TBil, AST, ALP, GGT, ALT, TC, TG, HDL-C, and LDL-C were adjusted.

The fitted smooth curve and the generalized linear model demonstrated a nonlinear relationship between TBA and femoral neck BMD (**Figure 2**).

Multiple regression analysis revealed that (**Table 2**): in the unadjusted model, TBA were positively correlated with femoral neck BMD (*β* = 0.05, 95% CI: 0.05–0.06, *p* < 0.0001). After adjusting for confounders, this positive association persisted for both model 2 (*β* = 0.06, 95% CI: 0.05–0.07, *p* < 0.0001) and model 3 (*β* = 0.04, 95% CI: 0.04–0.05, *p* <0.0001). After conversion of TBA from continuous to categorical (quartile) variables, the femoral neck BMD was 0.06 g/cm^2^ higher in the model 3 subjects with the highest TBA quartile than in the subjects with the lowest TBA quartile. The trend test between them was still significant based on the quartile of TBA (*p* < 0.0001).

**Table 2:** The association between TBA (µmol/L) and femoral neck BMD (g/cm^2^).

Parameters	Model 1	Model 2	Model 3
Quartiles of total bile acids			
Q1 (7.17–7.81 µmol/L)	0 (Reference)	0 (Reference)	0 (Reference)
Q2 (8.13–8.35 µmol/L)	0.04 (0.03, 0.05) <0.0001	0.05 (0.03, 0.06) <0.0001	0.03 (0.02, 0.04) <0.0001
Q3 (8.58–8.83 µmol/L)	0.08 (0.07, 0.09) <0.0001	0.08 (0.07, 0.09) <0.0001	0.06 (0.05, 0.07) <0.0001
Q4 (9.11–9.52 µmol/L)	0.11 (0.10, 0.12) <0.0001	0.11 (0.10, 0.13) <0.0001	0.09 (0.08, 0.11) <0.0001
*p* for trend	<0.0001	<0.0001	<0.0001
Total bile acids	0.08 (0.07, 0.09) <0.0001	0.09 (0.08, 0.09) <0.0001	0.07 (0.06, 0.08) <0.0001

Model 1 does not adjust covariates. Model 2: age and sex. Model 3: age, sex, education, obesity, smoking status, CKD, diabetes, hypertension, drinking status, TBil, AST, ALP, GGT, ALT, TC, TG, HDL-C, and LDL-C.

### Subgroup Analysis

**Figure 3:**
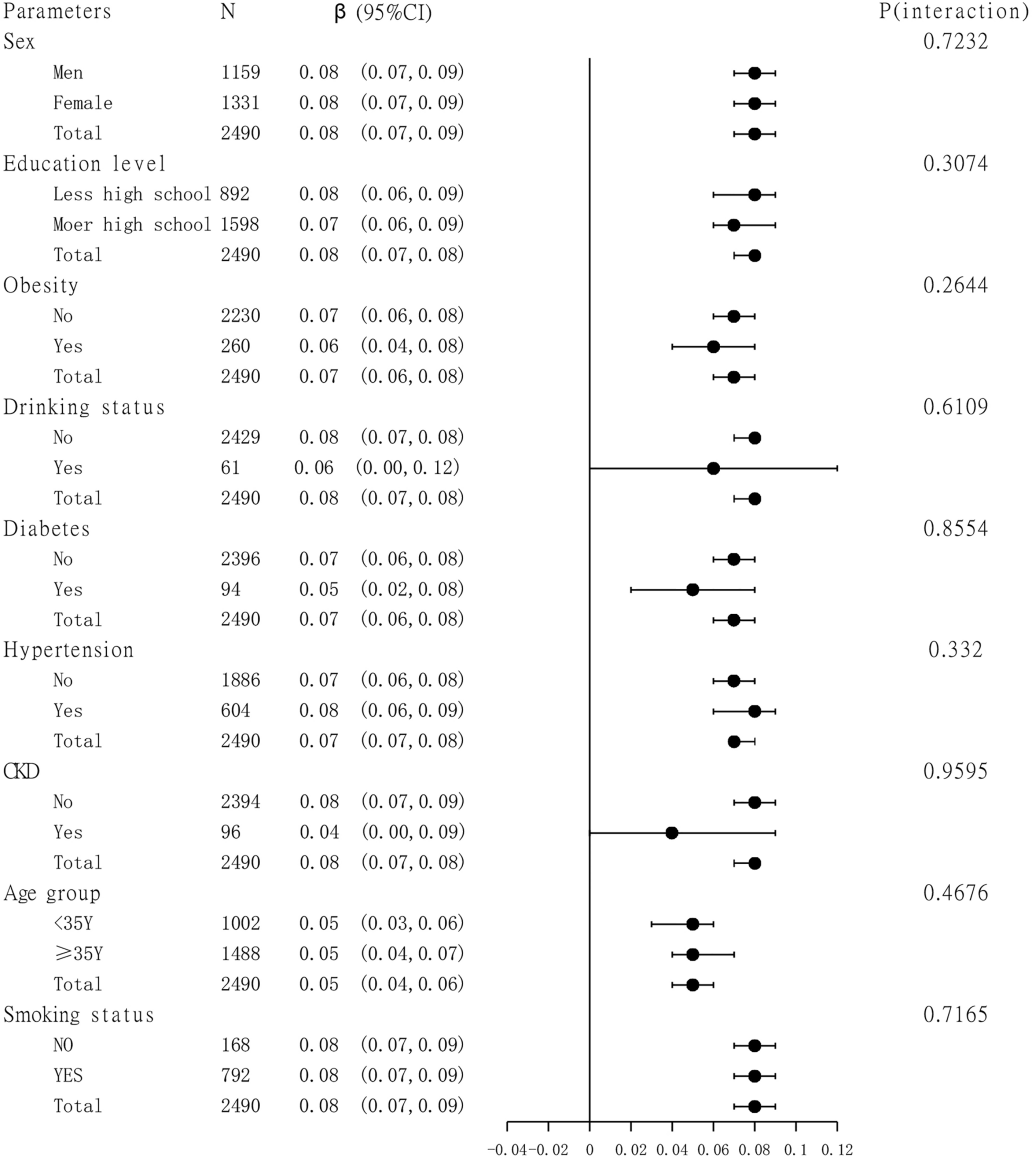
Subgroup analysis. TBil, AST, ALP, GGT, ALT, TC, TG, HDL-C, and LDL-C were adjusted.

A subgroup analysis of the association between TBA and femoral neck BMD is presented in **Figure 3**. Sex, age group (age is divided into <35 and ≥35 years), education level, smoking status, drinking status, obesity, hypertension, diabetes, and CKD were analyzed by subgroup analysis. The results showed a stable correlation between serum bile acids and femoral neck BMD in different subgroups.

## Discussion

This cross-sectional study of 2,490 Chinese people aged 20 to 59 years discovered an association between TBA and femoral neck BMD. Our study is the first to prove the correlation between TBA and femoral neck BMD in the young and middle-aged Chinese population aged 20 to 59 years, which has important clinical significance for bile acid metabolism and the prevention of osteoporosis.

Osteoporosis is known to be the most common skeletal disorder affecting the health of the population, and BMD is an important indicator for the diagnosis of osteoporosis (17). Assessing the factors associated with bone density can help with skeletal health guidance and education. Our study showed a positive correlation between TBA and BMD of the femoral neck in another Chinese postmenopausal woman, and serum bile acid levels in postmenopausal women were positively correlated with BMD in healthy postmenopausal women (18). This is similar to our results. Zhao et al. performed metabolite profiling on the serum of 136 Caucasian American women between the ages of 20 and 40 years and finally showed that serum bile acid levels were positively correlated with BMD (19). The specific mechanism of the association between serum bile acid levels and BMD is unknown. Bile acids, an important component of bile, play a significant role in the regulation of intestinal flora (6, 7, 20). The liver converts cholesterol into bile acids, which enter the intestine through the enterohepatic circulation and interact with the intestinal flora to affect the metabolism of the host (21). Among them, metabolites of bile acid receptors for secondary bile acids are expressed in immune cells, and the close interaction between these immune cells and bone cells regulates bone health and affects bone metabolism and bone density (22). Bone formation and absorption are also associated with bile acids, and in a serum bone turnover mark and metabolism-related study, type I collagen c-terminal peptide (a sensitive indicator of bone formation and absorption) was positively correlated with bile acid concentration (23). A number of studies have shown that the FXR, a receptor for bile acids, can inhibit osteoclastogenesis and promote osteoblastic formation (14, 24, 25). A previous study showed that *in vivo* deletions of FXR (FXR−/−) *in vivo* resulted in significant decreases in osteoclast count, osteoclast surface, and BMD in 8–20-week-old mice compared with FXR+/+ (26). The breakdown product of bile acids, chenodeoxycholic acid (CDCA), which can activate FXR, raising the alkaline phosphatase activity and calcification of ECM, provokes the expression of osteoblast marker genes (bone sialoprotein (BSP), osteocalcin (OC), osteopontin (OPN), and alkaline phosphate (ALP)), along with the activity of the bone transcription factor RUNX2 DNA-binding activity (27). Vitamin D is a fat-soluble vitamin that makes a valuable contribution towards calcium regulation and bone health (28). Bile acids, as the ligands of physiological vitamin D, promote the expression of the calcium transporter Trpv6 in the small intestine and increase the plasma calcium level (29). Another study has shown that a single bile acid, e.g., glycocholic acid (GCA), combined with vitamin D, can increase intestinal calcium uptake (30).

The current evidence for the association between TBA and BMD is limited and highly controversial. In a study of 148 children with chronic liver disease, BMD was not associated with bile acid levels after age, height, and weight adjustments (31). Results of another study in children with cholestasis associated with Alagille syndrome found a negative correlation between dual-energy X-ray absorptiometry (DXA) z-scores and serum bile acid levels (32). These findings may be due to the fact that children with cholestasis have severely elevated serum bile acid levels but less bile flowing into the hepatic and intestinal circulation, resulting in poor bone health associated with malabsorption and fat-soluble vitamin deficiency (32, 33).

This study population was a young and middle-aged population, and the results of this study may provide a new perspective on skeletal health guidance and education in the young and middle-aged population. In addition, the sufficient sample size allowed us to conduct further subgroup analysis. This is the main strength of this study. However, there are some limitations to our study. First, we only collected data from the general physical examination population aged 20–59 years old and did not analyze the relationship between serum bile acids and BMD among other age groups and constitutional populations. Second, due to the cross-sectional study design, we were unable to conclude a causal relationship between risk factors and osteoporosis. Third, it was a retrospective analysis, and a lack of information on participants’ menstrual conditions, fracture history, osteoporosis history, taking glucocorticoids, calcium supplementation, and vitamin D might have influenced the results of the study. Fourth, our study suggests that single-center results may not be generalizable. Therefore, further multicenter prospective studies and intervention strategies are needed to validate our results.

In conclusion, this study reports the positive association between total bile acids and femoral neck BMD in a young and middle-aged Chinese population. These results may reveal the important role of bile acids in bone health and bone metabolism in humans and provide evidence for early prevention and identification of low/decreased bone mineral density and risk factors for osteoporosis.

## Data Availability Statement

The original contributions presented in the study are included in the article/supplementary material. Further inquiries can be directed to the corresponding author.

## Ethics Statement

The studies involving human participants were reviewed and approved by the medical ethics committee of Hainan Western Central Hospital. Written informed consent for participation was not required for this study in accordance with the national legislation and the institutional requirements.

## Author Contributions

QL contributed to the conception and design of the research. JL and YC acquired the data from the database of Hainan Western Central Hospital in China. All authors contributed to the article and approved the submitted version.

## Conflict of Interest

The authors declare that the research was conducted in the absence of any commercial or financial relationships that could be construed as a potential conflict of interest.

## Publisher’s Note

All claims expressed in this article are solely those of the authors and do not necessarily represent those of their affiliated organizations, or those of the publisher, the editors and the reviewers. Any product that may be evaluated in this article, or claim that may be made by its manufacturer, is not guaranteed or endorsed by the publisher.

## References

[1] Leslie WD Morin SN. Osteoporosis Epidemiology 2013: Implications for Diagnosis, Risk Assessment, and Treatment. Curr Opin Rheumatol (2014) 26:440–6. doi: 10.1097/BOR.0000000000000064 24807402

[2] Varacallo MA Fox EJ. Osteoporosis and Its Complications. Med Clin North Am (2014) 98:817–31, xii-xiii. doi: 10.1016/j.mcna.2014.03.007 24994054

[3] Cui Z Meng X Feng H Zhuang S Liu Z Zhu T. Estimation and Projection About the Standardized Prevalence of Osteoporosis in Mainland China. Arch Osteoporos (2019) 15:2. doi: 10.1007/s11657-019-0670-6 31811461

[4] Zhang ZQ Ho SC Chen ZQ Zhang CX Chen YM. Reference Values of Bone Mineral Density and Prevalence of Osteoporosis in Chinese Adults. Osteoporos Int (2014) 25:497–507. doi: 10.1007/s00198-013-2418-2 23800746

[5] Zhu H Fang J Luo X Yu W Zhao Y Li X. A Survey of Bone Mineral Density of Healthy Han Adults in China. Osteoporos Int (2010) 21:765–72. doi: 10.1007/s00198-009-1010-2 19597908

[6] Chávez-Talavera O Tailleux A Lefebvre P Staels B. Bile Acid Control of Metabolism and Inflammation in Obesity, Type 2 Diabetes, Dyslipidemia, and Nonalcoholic Fatty Liver Disease. Gastroenterology (2017) 152:1679–94.e3. doi: 10.1053/j.gastro.2017.01.055 28214524

[7] Duparc T Plovier H Marrachelli VG Van Hul M Essaghir A Ståhlman M. Hepatocyte MyD88 Affects Bile Acids, Gut Microbiota and Metabolome Contributing to Regulate Glucose and Lipid Metabolism. Gut (2017) 66:620–32. doi: 10.1136/gutjnl-2015-310904 PMC5529962 27196572

[8] Fiorucci S Distrutti E. Bile Acid-Activated Receptors, Intestinal Microbiota, and the Treatment of Metabolic Disorders. Trends Mol Med (2015) 21:702–14. doi: 10.1016/j.molmed.2015.09.001 26481828

[9] Jacobs ET Haussler MR Alberts DS Kohler LN Lance P Martínez ME. Association Between Circulating Vitamin D Metabolites and Fecal Bile Acid Concentrations. Cancer Prev Res (Phila) (2016) 9:589–97. doi: 10.1158/1940-6207.CAPR-16-0033 PMC4993194 27138789

[10] Holick MF. Vitamin D Deficiency. N Engl J Med (2007) 357:266–81. doi: 10.1056/NEJMra070553 17634462

[11] Reid IR. Vitamin D Effect on Bone Mineral Density and Fractures. Endocrinol Metab Clin North Am (2017) 46:935–45. doi: 10.1016/j.ecl.2017.07.005 29080644

[12] Turner AG Anderson PH Morris HA. Vitamin D and Bone Health. Scand J Clin Lab Invest Suppl (2012) 243:65–72. doi: 10.3109/00365513.2012.681963 22536765

[13] Zheng T Kim NY Yim M. Fexaramine Inhibits Receptor Activator of Nuclear Factor-κb Ligand-Induced Osteoclast Formation *via* Nuclear Factor of Activated T Cells Signaling Pathways. J Bone Metab (2017) 24:207–15. doi: 10.11005/jbm.2017.24.4.207 PMC5734945 29259959

[14] Li Z Huang J Wang F Li W Wu X Zhao C. Dual Targeting of Bile Acid Receptor-1 (TGR5) and Farnesoid X Receptor (FXR) Prevents Estrogen-Dependent Bone Loss in Mice. J Bone Miner Res (2019) 34:765–76. doi: 10.1002/jbmr.3652 30536462

[15] Flegal KM Ogden CL Fryar C Afful J Klein R Huang DT. Comparisons of Self-Reported and Measured Height and Weight, BMI, and Obesity Prevalence From National Surveys: 1999-2016. Obes (Silver Spring) (2019) 27:1711–9. doi: 10.1002/oby.22591 PMC7289317 31544344

[16] Levey AS Coresh J Greene T Stevens LA Zhang YL Hendriksen S. Using Standardized Serum Creatinine Values in the Modification of Diet in Renal Disease Study Equation for Estimating Glomerular Filtration Rate. Ann Intern Med (2006) 145:247–54. doi: 10.7326/0003-4819-145-4-200608150-00004 16908915

[17] Camacho PM Petak SM Binkley N Diab DL Eldeiry LS Farooki A. American Association of Clinical Endocrinologists/American College of Endocinology Clinicla Practice Guidelines for the Diagnosis and Treatment of Postmenopausal Osteoporosis-2020 Update. Endocr Pract (2020) 26:1–46. doi: 10.4158/GL-2020-0524SUPPL 32427503

[18] Zhao YX Song YW Zhang L Zheng FJ Wang XM Zhuang XH. Association Between Bile Acid Metabolism and Bone Mineral Density in Postmenopausal Women. Clinics (Sao Paulo) (2020) 75:e1486. doi: 10.6061/clinics/2020/e1486 32187280 PMC7061317

[19] Zhao Q Shen H Su KJ Zhang JG Tian Q Zhao LJ. Metabolomic Profiles Associated With Bone Mineral Density in US Caucasian Women. Nutr Metab (Lond) (2018) 15:57. doi: 10.1186/s12986-018-0296-5 30116286 PMC6086033

[20] Molinaro A Wahlström A Marschall HU. Role of Bile Acids in Metabolic Control. Trends Endocrinol Metab (2018) 29:31–41. doi: 10.1016/j.tem.2017.11.002 29195686

[21] Wahlström A Sayin SI Marschall HU Bäckhed F. Intestinal Crosstalk Between Bile Acids and Microbiota and Its Impact on Host Metabolism. Cell Metab (2016) 24:41–50. doi: 10.1016/j.cmet.2016.05.005 27320064

[22] D’Amelio P Sassi F. Gut Microbiota, Immune System, and Bone. Calcif Tissue Int (2018) 102:415–25. doi: 10.1007/s00223-017-0331-y 28965190

[23] Bellissimo MP Roberts JL Jones DP Liu KH Taibl KR Uppal K. Metabolomic Associations With Serum Bone Turnover Markers. Nutrients (2020) 12:3161–14 doi: 10.3390/nu12103161 PMC7602719 33081124

[24] Id Boufker H Lagneaux L Fayyad-Kazan H Badran B Najar M Wiedig M. Role of Farnesoid X Receptor (FXR) in the Process of Differentiation of Bone Marrow Stromal Cells Into Osteoblasts. Bone (2011) 49:1219–31. doi: 10.1016/j.bone.2011.08.013 21893226

[25] Absil L Journé F Larsimont D Body JJ Tafforeau L Nonclercq D. Farnesoid X Receptor as Marker of Osteotropism of Breast Cancers Through Its Role in the Osteomimetism of Tumor Cells. BMC Cancer (2020) 20:640. doi: 10.1186/s12885-020-07106-7 32650752 PMC7350202

[26] Cho SW An JH Park H Yang JY Choi HJ Kim SW. Positive Regulation of Osteogenesis by Bile Acid Through FXR. J Bone Miner Res (2013) 28:2109–21. doi: 10.1002/jbmr.1961 23609136

[27] Caron S Huaman Samanez C Dehondt H Ploton M Briand O Lien F. Farnesoid X Receptor Inhibits the Transcriptional Activity of Carbohydrate Response Element Binding Protein in Human Hepatocytes. Mol Cell Biol (2013) 33:2202–11. doi: 10.1128/MCB.01004-12 PMC3648076 23530060

[28] Jin J. Screening for Vitamin D Deficiency in Adults. JAMA (2021) 325:1480. doi: 10.1001/jama.2021.4606 33847716

[29] Ishizawa M Akagi D Makishima M. Lithocholic Acid Is a Vitamin D Receptor Ligand That Acts Preferentially in the Ileum. Int J Mol Sci (2018) 19(7):1975. doi: 10.3390/ijms19071975 PMC6073204 29986424

[30] Casselbrant A Fändriks L Wallenius V. Glycocholic Acid and Butyrate Synergistically Increase Vitamin D-Induced Calcium Uptake in Caco-2 Intestinal Epithelial Cell Monolayers. Bone Rep (2020) 13:100294. doi: 10.1016/j.bonr.2020.100294 32715032 PMC7371747

[31] Loomes KM Spino C Goodrich NP Hangartner TN Marker AE Heubi JE. Bone Density in Children With Chronic Liver Disease Correlates With Growth and Cholestasis. Hepatology (2019) 69:245–57. doi: 10.1002/hep.30196 PMC6324969 30063078

[32] Kamath BM Ye W Goodrich NP Loomes KM Romero R Heubi JE. Outcomes of Childhood Cholestasis in Alagille Syndrome: Results of a Multicenter Observational Study. Hepatol Commun (2020) 4:387–98. doi: 10.1002/hep4.1468 PMC7049675 33313463

[33] Squires JE Celik N Morris A Soltys K Mazariegos G Shneider B. Clinical Variability After Partial External Biliary Diversion in Familial Intrahepatic Cholestasis 1 Deficiency. J Pediatr Gastroenterol Nutr (2017) 64:425–30. doi: 10.1097/MPG.0000000000001493 28045770

